# Trends and epidemiology in robotic‐assisted total knee arthroplasty: Reduced complications and shorter hospital stays

**DOI:** 10.1002/ksa.12353

**Published:** 2024-07-17

**Authors:** David Maman, Lior Laver, Roland Becker, Loai Ahmad Takrori, Assil Mahamid, Binyamin Finkel, Hadar Gan‐Or, Yaniv Yonai, Yaron Berkovich

**Affiliations:** ^1^ Department of Orthopedics Carmel Medical Center Haifa Israel; ^2^ Rappaport Faculty of Medicine Technion University Hospital (Israel Institute of Technology) Haifa Israel; ^3^ Department of Orthopedics Hillel Yaffe Medical Center Hadera Israel; ^4^ Department of Orthopedics and Traumatology University Hospital Brandenburg Berlin Germany

**Keywords:** national inpatient sample, post‐operative complications, robotic knee surgery, total knee arthroplasty

## Abstract

**Introduction:**

This study provides an in‐depth analysis of the immediate postoperative outcomes and implications or robotic‐assisted total knee arthroplasty (RA‐TKA) compared with conventional TKA (C‐TKA), particularly with regard to mortality, complications, hospital stay and costs, drawing from a comprehensive nationwide data set.

**Methods:**

The Nationwide Inpatient Sample (NIS) database, the largest all‐payer inpatient healthcare database in the United States, was used to identify all patients who underwent RA‐TKA or C‐TKA from 2016 to 2019. A total of 527,376 cases, representing 2,638,679 patients who underwent elective TKA were identified, of which 88,415 had RA‐TKA. To mitigate potential variations and selection bias in baseline characteristics between the two groups, a propensity score‐matched analysis was employed to further balance and refine our data set, resulting in 176,830 patients evenly distributed between the groups. Analysis was performed according to demographics, immediate post‐operative complications, and economic data, including payor class, length of stay and total charges.

**Results:**

There was a marked shift towards RA‐TKA, from an initial 0.70% in 2016 to a notable 7.30% by 2019. Patients who underwent RA‐TKA were slightly younger (66.2 ± SD years), compared to the C‐TKA group (66.7 ± SD years). Hospital stay was 1.89 days and 2.29 days for RA‐TKA and C‐TKA, respectively. Charges metrics revealed slightly higher charges for RA‐TKA. Less postoperative complications were found in the RA‐TKA group, such as blood loss, anaemia, acute kidney injury, venous thromboembolism, pulmonary embolism, pneumonia and surgical wound complication. Even following the propensity score matching, these findings remained consistent and statistically significant.

**Conclusions:**

RA‐TKA use in the United States has grown substantially in the last few years and has been associated with significantly reduced immediate post‐operative complications and length of hospital stay compared to C‐TKA, offering safer surgical management for TKA patients. Further studies on the short‐ and long‐term outcomes of RA‐TKA would improve the understanding of the full potential of this technology.

**Levels of Evidence:**

Level III.

**Abbreviations**
C‐TKA: conventional total knee arthroplasty
HCUP: Healthcare Cost and Utilization Project
ICD‐10: International Classification of Diseases, 10th Revision
KSS: Knee Society Score
LOS: length of stay
NIS: Nationwide Inpatient Sample
RA‐TKA: robotic‐assisted total knee arthroplasty
SPSS: Statistical Package for the Social Sciences
SSI: surgical site infection
TKA: total knee arthroplasty
WOMAC: Western Ontario and McMaster Universities Osteoarthritis Index

## INTRODUCTION

Robotic‐assisted total knee arthroplasty (RA‐TKA) has been increasingly used in orthopaedic surgery worldwide. Studies indicate potential long‐term advantages, such as faster recovery and enhanced knee mobility, due to the higher precision of alignment achieved with RA‐TKA [10, 13, 15, 22, 32, 33]. A systematic review reported superior clinical outcomes based on WOMAC and KSS in favour of RA‐TKA, though these results are based on a small number of studies [39]. Despite these promising outcomes, the need for RA‐TKA and its full benefits continue to be discussed.

Assessing RA‐TKA requires evaluating not only clinical outcomes but also immediate, short‐term benefits like postoperative complications, hospital costs, and length of stay. In 2011, knee arthroplasty accounted for 718,000 hospitalizations and 4.6% of all operating room procedures in the United States alone, highlighting its significant financial burden, particularly regarding the perioperative period [26, 37]. Thus, the assessment of early post‐operative complications remains an important aspect of TKA.

The number of TKAs has increased constantly throughout the last decades and it will likely continue to do so as life expectancy continues to increase. It has been suggested that by 2030, the demand for TKA will increase to 3.48 million surgeries annually in the United States [7]. Despite numerous advancements in TKA reflected by implant design, materials, recovery programs, pain management, reduction in blood loss, and thromboembolic and antibiotic prophylaxis, up to 20% of patients remain dissatisfied following TKA [1, 18, 28].

The latest evolutions in TKA have focused on advancements in surgical technique by improving accuracy in surgery and respecting patients' anatomy better. Given that surgeon‐controlled variables such as implant positioning, balanced flexion‐extension gaps, ligament tensioning and soft tissue preservation, alongside implant stability and survivorship, are pivotal for clinical outcomes, technological advancements facilitating the precise and reproducible achievement of these technical objectives may significantly enhance TKA outcomes [8, 9, 13, 24, 38].

It is hypothesized that achieving higher precision in planning and executing TKA surgery could lead to a less traumatic procedure. This, in turn, may result in fewer immediate post‐operative complications, shorter hospital stays and ultimately, reduced costs.

## MATERIALS AND METHODS

### Data set acquisition and inclusion criteria

This study utilized a comprehensive data set extracted from the Nationwide Inpatient Sample (NIS) [11], the largest publicly available all‐payer inpatient care database in the United States. Each entry in the data set, referred to as a ‘case’, represented a group of five patients meticulously matched based on general parameters. The resulting data set, extracted from the most recent version of the NIS, comprised 527,376 cases of TKA, encompassing a cohort of 2,638,679 patients. Notably, the NIS discharge weight indicates that each case extrapolates to five patients. Within this cohort, 88,415 patients underwent RA‐TKA, constituting 3.4% of the total patient population.

### Study period and data source

Spanning from 1 January 2016, to 31 December 2019, the data set represents the latest available information within the NIS system at the time of this study. The NIS, a core component of the Healthcare Cost and Utilization Project (HCUP) [7], captures 20% of inpatient stays from HCUP‐associated hospitals, amounting to approximately 7 million unweighted enrolments annually.

### Patient identification and exclusions

Patients undergoing TKA were identified based on ICD‐10 coding related to total knee replacement, with a comprehensive list of included codes available in the appendix. Exclusions comprised patients with non‐elective admissions or those who underwent surgery before admission.

### Statistical analyses and propensity score matching

Statistical analyses, including crosstabs and independent sample *t* tests, were conducted using SPSS 26 and MATLAB 2018 to compare RA‐TKA with C‐TKA. A significance level of *p* < 0.05 was applied. To address potential variations and selection bias, a propensity score‐matched analysis was performed using MATLAB 2018. The refined data set included 34,206 cases (representing 176,830 patients) with comparable characteristics undergoing either RA‐TKA or C‐TKA. Matching was based on various factors, including hospital size, patient location (urban‐rural code), median household income, region of the hospital and the total number of discharges from the hospital in the NIS data set.

### Comorbidity identification and data analysis

Comorbidities were identified through a review of patient‐specific ICD‐10 codes, with cases reporting hospital costs of $0 excluded. Analytical studies were conducted using SPSS 26 and Microsoft Excel to visualize annual cases, discern trends, and derive key statistical insights.

### Outcome measures and procedure identification

RA‐TKA procedures were delineated using specific ICD‐10‐PCS codes provided in the appendix. Clinical outcomes, including in‐hospital mortality, length of stay, complications and overall hospitalization costs, were analysed using established methodologies.

## RESULTS

**Figure 1:**
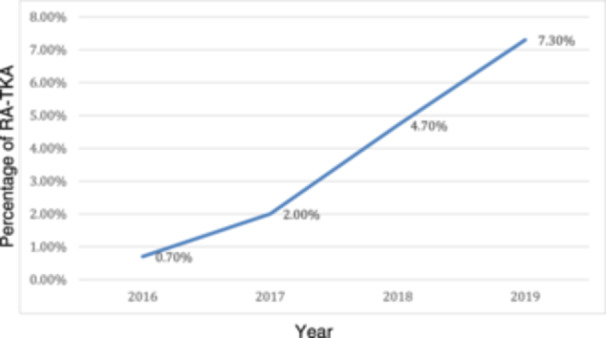
Percentage of robotic‐assisted total knee arthroplasty (RA‐TKA) based on the total number of TKA procedures (2016–2019). This figure illustrates the percentage of RA‐TKA procedures relative to the total number of TKA procedures performed annually between 2016 and 2019.

Within the extensive NIS data set of 527,376 cases, representing 2,638,679 patients, a remarkable increase in RA‐TKA was noticed. A total of 88,415 patients underwent RA‐TKA, comprising 3.4% of the total number of procedures. In 2016, RA‐TKA procedures were performed in only 5330 cases (0.7%) of all TKAs and increased to 39,495 cases (7.3%) by 2019 (Figure 1).

A comparative analysis of C‐TKA and RA‐TKA across age, payer distribution and gender is presented in Table 1. The average age at the time of surgery was 66.2 ± 9.46 years for the RA‐TKA patients and 66.7 ± 9.48 years for the C‐TKA.

**Table 1:** Comparative analysis of conventional total knee arthroplasty (C‐TKA) and robotic‐assisted total knee arthroplasty (RA‐TKA) across age, payer distribution and gender.

Parameter	RA‐TKA	C‐TKA	Significance
Total surgeries (%)	3.4	96.6	
Average age (years)	66.23	66.7	*p* < 0.001
Female (%)	58.8	61.6	*p* < 0.001
Payer—Medicare (%)	54.4	57.2	*p* < 0.001
Payer—Medicaid (%)	3.1	4.3	
Payer—private (%)	38.2	34.8	
Payer—other (including self‐pay) (%)	4.2	3.7	

*Note*: This table provides a comparative analysis of C‐TKA and RA‐TKA across various demographic and healthcare payer parameters.

The payer distribution varies significantly, with a higher percentage of Medicare and Medicaid payers in the C‐TKA group at 57.2% and 4.3%, respectively, compared to 54.4% and 3.1%, respectively, in the RA‐TKA group. The proportion of private payers was higher in the RA‐TKA group) 38.2% (in comparison to the C‐TKA group (34.8%).

The prevalence of various comorbidities in the RA‐TKA and C‐TKA groups is shown in Table 2. C‐TKA demonstrates a higher prevalence of the condition's dyslipidemia, sleep apnoea, chronic anaemia, alcohol abuse, osteoporosis, mental disorders, type 2 diabetes, renal disease and chronic lung disease.

**Table 2:** Prevalence of comorbidities in patients undergoing RA‐TKA or C‐TKA.

	RA‐TKA (%)	C‐TKA (%)	Significance
Hypertension diagnosis	57.6	59.4	*p* < 0.0001
Dyslipidemia diagnosis	44.1	46.6	*p* < 0.0001
Sleep apnoea diagnosis	13.8	13.2	*p* < 0.0001
Chronic anaemia	4.9	5.9	*p* < 0.0001
Alcohol abuse	0.7	0.9	*p* < 0.0001
Osteoporosis	3.7	4	*p* < 0.0001
Mental disorders	27.9	29	*p* < 0.0001
Parkinson disease	0.5	0.6	*p* = 0.191
Type 2 diabetes	19.2	21.6	*p* < 0.0001
Renal disease	5.5	7.1	*p* < 0.0001
Congestive heart failure	1.2	1.3	*p* = 0.017
Chronic lung disease	5.0	6.0	*p* < 0.0001

*Note*: This table displays the prevalence of common comorbidities among patients who underwent either RA‐TKA or C‐TKA.

Abbreviations: C‐TKA, conventional total knee arthroplasty; RA‐TKA, robotic‐assisted total knee arthroplasty.

In order to overcome potential selection bias and baseline differences in terms of existing comorbidities, a propensity score‐matched analysis was performed, ensuring that the two groups compared are statistically equivalent, thus minimizing selection bias. Propensity score‐matched analysis is a statistical method that helps compare two groups in observational studies. It balances participant characteristics by pairing individuals with similar likelihoods of being in either group, making the comparison more reliable and reducing the impact of confounding variables. This approach aims to emulate the random assignment seen in experiments, improving the accuracy of conclusions drawn from non‐randomized studies. It offers insights into demographics, payer details, and the prevalence of various medical conditions, demonstrating the average age, gender distribution, and type of payer, alongside an array of diagnoses, in both the RA‐TKA and C‐TKA groups. The propensity score‐matched analysis data is presented in Table 3. No significant disparities were discerned between the groups in most of the examined parameters, highlighting the homogeneous nature of the two patient cohorts and underlining the effectiveness and reliability of the applied propensity score‐matched analysis.

**Table 3:** Comparison of demographic and clinical data in propensity score‐matched cohorts of C‐TKA and RA‐TKA.

Parameter	RA‐TKA (%)	C‐TKA (%)	Significance
Total surgeries (number)	88,415	88,415	
Average age (years)	66.23	66.34	*p* = 0.062
Female (%)	58.8	59.5	*p* = 0.147
Payer—Medicare (%)	54.4	55.4	*p* = 0.198
Payer—Medicaid (%)	3.1	3.3	
Payer—private (%)	38.2	37.5	
Payer—other (including self‐pay) (%)	4.2	3.8	
Hypertension diagnosis	57.6	58	*p* = 0.18
Dyslipidemia diagnosis	44.1	44.6	*p* = 0.219
Sleep apnoea diagnosis	13.8	13.4	*p* = 0.068
Chronic anaemia	4.9	4.6	*p* = 0.211
Alcohol abuse	0.7	0.7	*p* = 0.85
Osteoporosis	3.7	3.4	*p* = 0.075
Mental disorders	27.9	27.8	*p* = 0.923
Parkinson disease	0.5	0.4	*p* = 0.19
Type 2 diabetes	19.2	19.2	*p* = 0.891
Renal disease	5.5	5.4	*p* = 0.702
Chronic heart failure	1.2	1	*p* = 0.074
Chronic lung disease	5	4.6	*p* = 0.116

*Note*: This table presents a comparison of demographic and clinical data between propensity score‐matched cohorts of patients who underwent C‐TKA and RA‐TKA.

Abbreviations: C‐TKA, conventional total knee arthroplasty; RA‐TKA, robotic‐assisted total knee arthroplasty.

The results in hospitalization time, post‐procedure, and comparison are shown in Table 4 between RA‐TKA and C‐TKA. No significant difference was observed in the mortality rate (*p* = 1.000). However, significant differences were found in both the average length of stay and the mean total charges in favour of RA‐TKA (*p* < 0.0001).

**Table 4:** Comparison of hospitalization outcomes in propensity score‐matched cohorts of C‐TKA and RA‐TKA.

	RA‐TKA (%)	C‐TKA (%)	Significance
Died during hospitalization	0.02%	0.02%	*p* = 1
Length of stay mean in days	1.89 (Std. deviation 1.70)	2.41 (Std. deviation 1.41)	*p* < 0.0001
Total charges mean in $	65,891 (Std. deviation 41,042)	62,464 (Std. deviation 38,625)	*p* < 0.0001

*Note*: This table compares hospitalization outcomes between propensity score‐matched cohorts of patients who underwent C‐TKA and RA‐TKA.

Abbreviations: C‐TKA, conventional total knee arthroplasty; RA‐TKA, robotic‐assisted total knee arthroplasty.

Table 5 presents a comparison of postoperative complications between robotic and non‐robotic surgeries after applying propensity score‐matching to ensure a balanced comparison. Noteworthy are the significant differences in various complications, such as anaemia, acute kidney failure, venous thromboembolism, pulmonary embolism, pneumonia and surgical wound complication, which had significantly lower rates in the RA‐TKA group.

**Table 5:** Comparison of postoperative complications in propensity score‐matched cohorts of RA‐TKA and C‐TKA.

	RA‐TKA (%)	C‐TKA (%)	Significance
Anaemia	11.508	17.073	*p* < 0.0001
Acute kidney injury	1.318	1.602	*p* = 0.016
Heart failure	0.096	0.064	*p* = 0.257
Acute coronary artery disease	0.057	0.036	*p* = 0.317
Stroke	0.006	0.006	*p* = 1
Pulmonary oedema	0.028	0.058	*p* = 0.197
Venous thromboembolism	0.187	0.327	*p* = 0.010
Pulmonary embolism	0.074	0.228	*p* < 0.0001
Pneumonia	0.085	0.181	*p* = 0.018
Surgical wound complication	0.0283	0.5672	*p* < 0.0001

*Note*: This table presents a comparison of postoperative complications between propensity score‐matched cohorts of patients who underwent RA‐TKA and C‐TKA.

Abbreviations: C‐TKA, conventional total knee arthroplasty; RA‐TKA, robotic‐assisted total knee arthroplasty.

## DISCUSSION

The main finding of this study is that RA‐TKA is associated with significantly reduced early post‐operative complications and length of hospital stay compared to C‐TKA, albeit with slightly higher costs. The rapid adoption of RA‐TKA from 2016 to 2019 demonstrates the increasing acceptance of this technology in clinical practice.

The results of this study are consistent with previous research indicating that RA‐TKA improves surgical precision and patient outcomes [2, 10, 25, 29, 30, 31, 32, 35]. The reduced incidence of complications such as anaemia [36], acute kidney injury, venous thromboembolism, pulmonary embolism, pneumonia, and surgical wound complications in the RA‐TKA group highlights the potential benefits of robotic assistance in reducing surgical trauma and improving recovery [23].

The shorter LOS for RA‐TKA patients (1.89 days) compared to C‐TKA patients (2.41 days) is clinically significant, suggesting that RA‐TKA may facilitate faster recovery and support the concept of outpatient TKA surgery, which is gaining popularity [6, 13, 29, 31]. This reduction in hospital stay is particularly important in the context of increasing healthcare costs and the need for efficient resource utilization.

Despite the higher initial costs associated with RA‐TKA [27] ($65,891 vs. $62,464 for C‐TKA), the reduction in post‐operative complications and shorter hospital stays may offset these costs in the long term [5, 16]. The economic benefits of RA‐TKA could be further explored in future studies, considering the potential savings from reduced readmissions and complications.

Several recent studies support the findings of this study. For instance, Cheng et al. [3] found that robotic‐assisted TKA is associated with the use of thinner polyethylene liners, potentially improving joint function and longevity. Similarly, Erard et al. [8] reported enhanced soft tissue balance with robotic‐assisted TKA, which may contribute to better clinical outcomes. Lee et al. [17] demonstrated that functional alignment maximizes the advantages of robotic arm‐assisted TKA with better patient‐reported outcomes compared to mechanical alignment. Kayani et al. [14] found that robotic‐arm‐assisted TKA is associated with improved forgotten joint scores at 5‐year follow‐up. These findings align with the observed reduction in complications and improved recovery in this study.

Furthermore, Choi et al. [4] demonstrated that functional alignment with robotic‐arm‐assisted TKA results in better patient‐reported outcomes than mechanical alignment with manual TKA. Turan et al. [34] reported coronal alignment improvement with robotic‐assisted techniques, although without significant clinical differences. McCormick et al. [22] showed that robotic‐assisted technology does not influence functional outcomes amongst obese and morbidly obese TKA patients, while Itou et al. [12] found no increased risk of postoperative deep vein thrombosis with robotic‐assisted TKA.

## LIMITATIONS

This study has several limitations. The NIS database provides extensive healthcare data, but it may contain errors due to suboptimal coding and manual entry. Additionally, the NIS only provides data for the in‐hospital period, limiting the ability to assess long‐term outcomes [19, 20, 21]. Future studies should include long‐term follow‐up to better understand the full impact of RA‐TKA on patient outcomes.

Another limitation is the potential selection bias, as the choice of surgical technique may be influenced by patient characteristics and surgeon preferences. Although propensity score matching was used to minimize this bias, unmeasured confounding factors may still affect the results. Further research should explore the impact of these factors on the outcomes of RA‐TKA.

Despite these limitations, the strengths of this study include the use of a large, nationally representative data set and the application of rigorous statistical methods to ensure robust and reliable results. The findings contribute to the growing body of evidence supporting the benefits of RA‐TKA in improving patient outcomes and reducing healthcare costs.

## CONCLUSION

RA‐TKA has shown substantial growth in usage and is associated with significantly reduced early post‐operative complications and shorter hospital stays compared to C‐TKA. Despite higher initial costs, RA‐TKA offers safer surgical management for TKA patients. Further studies on the short‐ and long‐term outcomes of RA‐TKA would improve the understanding of the full potential of this technology.

## AUTHOR CONTRIBUTIONS

David Maman: Conducted major parts of the work, data analysis and manuscript writing. Lior Laver: Manuscript writing. Roland Becker and Yaniv Yonai: Provided clinical expertise. Assil Mahamid: Contributed to manuscript revisions. Loai Ahmad Takrori: Assisted with data validation and analysis. Binyamin Finkel: Participated in data collection and preliminary analysis. Hadar Gan‐Or: Provided support in statistical analysis and validation. Yaron Berkovich: Conceived the study idea and mentored the project.

## CONFLICT OF INTEREST STATEMENT

The authors declare no conflicts of interest.

## ETHICS STATEMENT

The study was conducted under exempt status granted by the institutional review board, and the requirement for informed consent was waived due to the de‐identified nature of the NIS data set.

## Supporting information

Supporting information.

## Data Availability

The data that support the findings of this study are available from the National Inpatient Sample (NIS). However, restrictions apply to the availability of these data, which were used under license for the current study, and so are not publicly available. Data are available from the authors upon reasonable request and with permission of the NIS.
